# Clinical assessment of children with long COVID syndrome

**DOI:** 10.1038/s41390-022-02378-0

**Published:** 2022-12-07

**Authors:** Réka Garai, Péter Krivácsy, Vivien Herczeg, Fanni Kovács, Bálint Tél, Judit Kelemen, Anna Máthé, Eszter Zsáry, Johanna Takács, Dániel Sándor Veres, Attila J. Szabó

**Affiliations:** 1 1st Department of Pediatrics, Semmelweis University, Budapest, Hungary; 2 Semmelweis Pediatric Long COVID Research Group, Budapest, Hungary; 3 Centre for Translational Medicine, Semmelweis University, Budapest, Hungary; 4 Faculty of Medicine, Semmelweis University, Budapest, Hungary; 5 Department of Social Sciences, Faculty of Health Sciences, Semmelweis University, Budapest, Hungary; 6 Department of Biophysics and Radiation Biology, Semmelweis University, Budapest, Hungary; 7 ELKH-SE Pediatrics and Nephrology Research Group, Budapest, Hungary

## Abstract

**Background:**

There is a need for further understanding pediatric long COVID syndrome (LCS) to be able to create specific case definitions and guidelines for providing good clinical care.

**Methods:**

Medical records of all LCS patients who presented at our designated LC clinic were collected. We carried out descriptive analyses summarizing the history, clinical presentation, and findings of children, while doing a diagnosis of exclusion with multi-disciplinary medical examinations (physical, laboratory, and radiological examinations, specialist consultations, etc.) without a control group.

**Results:**

Most children reported at least minor impairment to their quality of life, of which 17 (23%) had moderate or severe difficulties. Findings that could be directly connected to the linked complaint category were observed in an average of 18%, respiratory symptoms with objective alterations being the most frequent (37%). Despite our detecting mostly non-specific conditions, in a smaller number we identified well-described causes such as autoimmune thyroiditis (7%).

**Conclusions:**

The majority of children stated an impairment in their quality of life, while symptom-related conditions were detected only in a minority. Controlled studies are needed to separate the effect of the pandemic era from the infection itself. Evidence-based pediatric guidelines could aid to rationalize the list of recommended examinations.

**Impact:**

- Long COVID syndrome is a complex entity with a great impact on children’s everyday lives. Still, there is no clear guidance for pediatric clinical management. Systematic, detailed studies with medical assessment findings could aid the process of creating evidence-based guidelines.
- We present validated systematic information collected during in-person medical assessments with detailed medical findings and quality of life changes.
- While making a diagnosis of exclusion, we could confirm symptom-related conditions only in a minority of children; however, the majority reported at least minor impairment to their quality of life.

## Introduction

Based on the considerable research conducted in the past 2 years, children have been indicated to have relatively lower rates of severe acute Coronavirus disease 2019 (COVID-19).^1^ However, the latest clinical experience shows that post-acute consequences of the infection could mean a risk of similarly impactful outcomes.^2,3^

The World Health Organization (WHO) developed a clinical case definition of post-COVID condition by Delphi consensus in October 2021; however, it is indicated that it might not be suitable for children.^4^ Estimating the epidemiological and medical characteristics of COVID-19-related diseases in children is difficult. Based on a meta-analysis published in August 2021, it is predicted that 35% of all cases remained asymptomatic throughout the infection.^5^ According to a recent British calculation, the prevalence of symptoms that remain 12 weeks after Coronavirus infection ranges from 3% based on tracking specific symptoms to 12% based on self-classification of long COVID syndrome (LCS; https://www.ons.gov.uk/peoplepopulationandcommunity/healthandsocialcare/conditionsanddiseases/articles/technicalarticleupdatedestimatesoftheprevalenceofpostacutesymptomsamongpeoplewithcoronaviruscovid19intheuk/26april2020to1august2021).

LCS is a growing health concern as its persisting and emerging symptoms are extensive, often overlapping, fluctuating, changing over time, and can affect multiple organ systems with a potentially negative impact on the quality of life.^6,7^ In the pediatric field, only a few prospective studies collecting systematic data in larger cohorts of children with multidisciplinary clinical assessment are available. They also often fail to include quality of life changes, detailed clinical characteristics, or confirmation of the acute infection.^8^ Other studies that followed up patients previously hospitalized at the acute phase of COVID are reporting answers given to interviews without clinical assessment.^9^ Studies focusing on the long-lasting neurological,^10^ cardiac,^11^ pulmonary,^12^ or psychological^13^ effects of COVID-19 infection are available, but only separately.

In February 2021, an increasing number of children presented at our Emergency Unit at the 1st Department of Pediatrics, Semmelweis University with permanent, often debilitating symptoms. We opened our LC outpatient clinic in March 2021 with the purpose of further investigating this condition and understanding the natural history of the disease.

Since the course of the illness seems to be different in children than in adults,^14^ we aim in our present case series to provide a deeper insight into pediatric LCS.

## Methods

### Data collection

In our observational study, patients underwent standard-of-care examinations, and blood samples were collected primarily for diagnostic purposes. Data was collected as a component of ambulatory care and stored in a standardized case report form (CRF), which included online surveys for patients and employed the WHO Post COVID CRF (https://www.who.int/publications/i/item/global-covid-19-clinical-platform-case-report-form-(crf)-for-post-covid-conditions-(post-covid-19-crf-)) for data collection. It consists of questions about the acute infection, lingering symptoms, and functioning and medical findings, which we extended and translated to match the institute-specific purposes. Responses were validated and additional outcomes were recorded by the physician in the CRF. We have been granted an ETT TUKEB ethical approval, the number of which is: IV/5943-1/2021/EKU.

### Case definition

Utilizing the National Institute for Clinical Excellence (NICE) guideline’s case definitions,^15^ LCS cases were defined based on the presence of specific symptoms and the clinical (for example, olfactory impairment) and/or laboratory evidence of COVID-19 at least 1 month preceding the current symptoms.

The guideline distinguishes three categories related to COVID-19; first, the “acute disease,” which is present for up to 4 weeks from first symptom onset. The “ongoing symptomatic” phase, which can be used for conditions reported after the acute infection, but not lasting longer than 12 weeks; and “post-COVID-19 syndrome,” which refers to symptoms and signs occurring since or after an infection related to COVID-19 continue for more than 12 weeks and are not explained by an alternative diagnosis. Symptoms (usually clusters of symptoms) can overlap, fluctuate, and change over time and can affect any organ system.^15^

The term “long COVID,” which we prefer to use, until the presence of a specific clinical case definition for children, includes both the “ongoing symptomatic” phase and “post-COVID syndrome.”^15^ To present as reliable data as possible, we excluded those from our analysis who met the criteria of the NICE case definition with a strong clinical suspicion of the acute infection but for whom we could not confirm the acute COVID with laboratory tests.

### Study sample and patient management

Data were collected during the visits undertaken at the 1st Department of Pediatrics from March 24 through May 26, 2021. Patients could make online appointments to our facility from across the country without restrictions. A general practitioner’s referral or a preassessment was not required. Before their first visit, parents filled out the online questionnaire with the help of their children regarding the general, acute COVID-19-specific, and LCS-specific history and persisting symptoms, as well as the quality of the children’s lives. The survey contained a total of 50 questions about their current complaints, which could be categorized into 10 major organ system categories (general, cardiovascular, neurologic, mental, gastrointestinal [GI], dermatologic, musculoskeletal, pulmonary, ear–nose–throat and ophthalmologic [ENT-O], and reproductive) as seen in Supplementary Table S1.

A Quality of Life (Functioning) (QoL-F) component was also included in the questionnaire, which was measured by 12 items on a 5-grade scale from “no difficulty” to “extreme difficulty/cannot do.” Furthermore, the QoL-F items were compared to the situation before COVID-19 on a 3-point scale (better, same, worse) to measure changes in QoL-F. The total scores of the QoL-F and QoL-F changes were converted to an index value between 0 and 100, taking into account the number of answered questions (QoL-F: 0—no difficulty, 100—extreme difficulty/cannot do; QoL-F changes: 0—better, 50—same, 100—worse).

Relying on the NICE guideline,^16^ all patients underwent physical examination and blood tests, with particular emphasis on the following parameters: complete blood count, clinical chemistry tests [ions, liver function (GOT, GPT, GGT, ALP), renal function (urea, creatinine), CRP, CK, protein, albumin, LDH, ferritin], IL-6, BNP, D-dimer, troponin, thyroid function (TSH, T3, T4), coagulation parameters, and autoantibodies specific for autoimmune thyroiditis (ATG, ATPO, TRAK) and COVID-19 IgG, IgM antibodies.

Further examinations (e.g., imaging) and consultations were indicated according to the symptoms and to the laboratory and physical findings.

### Standardized study definitions

Normal levels of laboratory values were defined by institutional age-specific pediatric standards. The presence of a medical condition was based on discharge diagnoses in previous medical documentation or discharge diagnoses given during our checkup. Obesity was determined based on national age-specific body mass index *Z* scores.^16^

The list and details of undertaken examination can be found in Tables 1–3 and Supplementary Tables S4–S6*.*

**Table 1:** The number of consultations, imaging, and further examinations based on organ-specific complaints with the rates of positive findings and matching complaints.

Number of patients with at least one group-specific COMPLAINT (*n*)	Examination	Positive findings/performed examinations (positive findings %)	Number of patients with at least one positive finding in the subgroup/number of patients with at least one group-specific complaint (matching complaints %)
GENERAL (*N* = 77)		21/85 (25%)	21/77 (27%)
	Neck US	7/9 (78%)	
	Thyroid screening	11/85 (12%)	
	6mWT^a^	10/29 (34%)	
NEUROLOGIC (*N* = 74)		5/44 (11%)	5/74 (7%)
	Neurological examination	5/43 (12%)	
	Head MRI^b^	2/11 (18%)	
	Spinal MRI	0/1 (0%)	
	CSF^c^	2/2 (100%)	
	EEG	0/7 (0%)	
MENTAL HEALTH (*N* = 64)		15/24 (63%)	15/64 (23%)
	Mental specialist consultation	15/24 (63%)	
CARDIOVASCULAR (*N* = 55)		8/44 (18%)	8/55 (15%)
	Echocardiography	5/39 (13%)	
	Cardiac MRI	0/1 (0%)	
	ECG	4/44 (9%)	
	Holter ECG	0/2 (0%)	
GASTROINTESTINAL (*N* = 53)		8/37 (22%)	8/53 (15%)
	Abdominal US	6/35 (17%)	
	Celiac disease screening	0/11 (0%)	
	Stool occult blood test	0/12 (0%)	
	Stool culture	0/10 (0%)	
	H_2_ breath test	2/2 (100%)	
EAR–NOSE–THROAT AND OPHTHALMOLOGY (*N* = 49)		8/41 (20%)	8/49 (16%)
	ENT specialist consultation	3/16 (19%)	
	Ophthalmology specialist	7/32 (22%)	
MUSCULOSKELETAL (*N* = 45)			
	Knee US	2/3 (67%)	
	Other imaging examinations^d^	1/2 (50%)	
PULMONARY (*N* = 30)		11/40 (28%)	11/30 (37%)
	Chest CT	0/3 (0%)	
	Pulmonary US	0/1 (0%)	
	Chest X-Ray^e^	4/33 (12%)	
	Lung function test	11/24 (46%)	
DERMATOLOGIC (*N* = 23)		5/8 (63%)	5/23 (22%)
	Dermatology specialist consultation	5/8 (63%)	
TOTAL		83/328 (25%)	83/465 (18%)

*6mWT* 6-min walking test, *CSF* cerebrospinal fluid, *CT* computed tomography, *ECG* electrocardiogram, *EEG* electroencephalogram, *MRI* magnetic resonance imaging, *US* ultrasound.

^a^Subject had to stop during the 6 min, or had a drop in SpO_2_ < 95%, or their heart rate elevated to 1.5× of their resting heart rate.

^b^Positive MRIs were considered potentially normal variants by agreement between a radiologist and a pediatric neurology specialist.

^c^Positive CSF samples showed only marginally elevated protein levels and were considered to be of low clinical importance by a pediatric neurology specialist.

^d^Hip US, shoulder US, sternocostal MRI.

^e^Chest X-Rays were performed because of pulmonary and/or cardiological reasons.

**Table 2:** Table of average laboratory findings.

Parameter	Unit	*N*	Altered, *n* (%)	Mean	Minimum	Maximum
Albumin	g/l	84	1 (1.2)	46.67	41.00	51.00
GPT	U/l	86	3 (3.5)	17.61	8.00	65.00
GOT	U/l	87	4 (4.6)	26.61	13.00	80.00
Protein	g/l	86	2 (2.3)	73.02	64.00	85.00
Blood urea nitrogen	mmol/l	87	2 (2.3)	4.62	1.50	7.50
Creatine kinase	U/l	81	9 (11.1)	126.04	27.00	924.00
Creatinine	μmol/l	87	25 (28.7)	47.79	24.00	86.00
Uric acid	μmol/l	30	1 (3.3)	236.70	103.00	346.00
Alkaline phosphatase	U/l	84	32 (38.1)	208.54	52.00	1413.00
CRP	mg/l	87	1 (1.1)	0.70	0.09	10.20
Ferritin	μg/l	73	46 (63.0)	29.18	1.50	82.00
Iron	μmol/l	72	14 (19.4)	16.48	4.00	36.00
Transferrin saturation	%	69	24 (34.8)	30.83	6.00	79.00
Lactate dehydrogenase	U/l	85	44 (51.8)	200.33	122.00	470.00
Leukocytes	G/l	86	19 (22.1)	6.74	3.00	20.40
Neutrophils	%	87	17 (19.5)	49.77	27.40	70.00
Monocytes	%	87	64 (73.6)	6.89	4.00	10.90
Lymphocytes	%	87	4 (4.6)	39.79	22.70	64.10
Red Blood Cells	T/l	87	7 (8.0)	4.80	3.90	5.60
Hemoglobin	g/l	87	14 (16.1)	135.29	103.00	161.00
Hematocrit	l/l	87	5 (5.7)	0.86	0.32	41.30
Platelets	U/l	87	1 (1.1)	288.07	173.00	440.00
Thyroid peroxidase antibody	U/ml	85	8 (9.4)	40.84	0.16	2381.12
Antithyroglobulin antibody	U/ml	85	7 (8.2)	98.98	10.00	4000.00
Thyroid-stimulating hormone	mU/l	87	4 (4.6)	2.40	0.71	9.54
IL-6	pg/ml	61	1 (1.6)	1.87	1.00	8.36
D-dimer	μg/ml	85	7 (8.2)	0.36	0.17	1.43
NT-pro-BNP	pg/ml	82	4 (4.4)	41.69	9.99	240.90
Troponin T	ng/l	86	0	3.38	0.00	13.00
**Cerebrospinal** **fluid 1**	**Unit**		**Cerebrospinal fluid 2**	**Unit**		
Protein	g/l	0.5	Protein	g/l	0.5	
Glucose	mmol/l	3.4	Glucose	mmol/l	3.7	
Cells wbc/B		3	Cells wbc/B		Negative

**Table 3:** List of undertaken examinations and positive findings.

Examination	Positive findings, *n* (%)^a^	Negative result, *n* (%)^a^	Unknown/incomplete data, *n* (%)^a^
Physical examination (*n* = 89)	45 (50.6)	44 (49.4)	0
Neurology (*n* = 8)	2 (25.0)	5 (62.5)	1 (12.5)
CSF (*n* = 2)^b^	2 (100)^b^	0	0
EEG (*n* = 7)	0	7 (100)	0
ENT-O (*n* = 41)	8 (19.5)	19 (46.3)	14 (34.1)
ENT specialist (*n* = 16)	3 (18.8)	8 (50)	5 (31.3)
Ophthalmology specialist (*n* = 33)	7 (21.2)	14 (42.4)	12 (36.4)
Radiology (*n* = 71)	20 (28.2)	46 (64.8)	5 (7.0)
Chest CT (*n* = 3)	0	3 (100)	0
Echocardiography (*n* = 39)	5 (12.8)	30 (76.9)	4 (10.3)
Lung US (*n* = 1)	0	1 (100)	0
Head MRI (*n* = 11)^c^	2 (18.2)^c^	8 (72.7)	1 (9.1)
Vertebral MRI (*n* = 1)	0	1 (100)	0
Abdominal US (*n* = 35)	6 (17.1)	29 (82.9)	0
Neck US (*n* = 9)	7 (77.8)	2 (22.2)	0
Chest X-ray (*n* = 33)	4 (12.1)	29 (87.9)	0
Knee US (*n* = 3)	2 (66.7)	1 (33.3)	0
Chest MRI (*n* = 1)	0	1 (100)	0
Cardiology (*n* = 53)	21 (39.6)	31 (58.5)	1 (1.9)
ECG (*n* = 44)	4 (9)	37 (84)	3 (6)
6mWT (*n* = 43)^d^	21 (48.8)^d^	13 (30.2)	9 (20.9)
Holter ECG (*n* = 4)	0	0	2 (100)
Pulmonology (*n* = 24)	12 (41.4)	12 (41.4)	5 (17.2)
Lung function test (*n* = 24)	11 (45.8)	9 (37.5)	4 (16.7)
Gastroenterology (*n* = 18)	2 (11.1)	12 (66.7)	4 (22.2)
Celiac screening (*n* = 11)	0	10 (90.9)	1 (9.1)
Stool occult blood test (*n* = 12)	0	7 (58.3)	5 (41.7)
Stool culture (*n* = 10)	0	3 (30.0)	7 (70.0)
H_2_ breath test (*n* = 2)	2 (100)	0	0
Dermatology (*n* = 8)	5 (62.5)	0	3 (37.5)
Mental health (*n* = 25)	15 (60)	10 (40)	0

^a^Percentages were calculated with the total numbers of each examination group.

^b^Positive CSF samples showed only marginally elevated protein levels and were considered to be of low clinical importance by a pediatric neurology specialist.

^c^Positive MRIs were considered potentially normal variants by agreement between a radiologist and a pediatric neurology specialist.

^d^Subject had to stop during the 6 min, or had a drop in SpO_2_ < 95%, or their heart rate elevated to 1.5× of their resting heart rate.

### Data extraction and statistical analysis

Data was extracted from the CRFs, stored in data tables, and re-validated before being used for data analysis with the help of professional biostatisticians. To describe the data, we used the distribution of relative frequencies and descriptive analysis. If not otherwise stated, data was presented as mean ± SD or absolute frequency and proportion. We used independent samples *t* test to examine sex differences with Hedges’ *g* effect size, confidence intervals, and Pearson’s correlation for associations between variables. The level of significance was set a priori at 0.05. Statistical analysis and visualization were conducted using IBM SPSS Statistics software, Version 25.0 (IBM Corp.), and *qgraph* package (version 1.6.9.) in R.^17^

## Results

### Demographics and SARS-COV-2 testing

From March 24 through May 26, 2021, a total of 103 children sought ambulatory care at our LCS clinic; of these, 6 did not fulfill the LCS criteria.^15^ We excluded another 8 children from the analysis, who clinically fulfilled the case definition, but we could not verify the presence of the acute infection, therefore we enrolled 89 in our final analysis.

Of the 89 patients with LCS, 33 (37.1%) were male. The mean age was 11.4 ± 3.6 years, lower in males than in females (10.1 vs. 12.3 years, *P* = 0.02, *g* = 0.60 [0.16; 1.03]). All patients belonged to the Caucasian ethnicity (Table 4).

**Table 4:** Demographics and symptom characteristics of children with LC.

Characteristics	Overall	(%)	Male	(%)	Female	(%)	*P*^Male vs. Female^	*g*/*r* [95% CI]
Ethnicity, *n*								
Caucasian	89	(100)	33	(37)	56	(63)		
Mean age, years (SD)	11.4	(3.8)	10.1	(4.2)	12.3	(3.2)	**0.015**	0.60 [0.16; 1.03]
Age groups, *n*								
0–5 years	6	(6)	6	(18)	0	(0)		
6–11 years	36	(40)	13	(40)	23	(41)		
12–18 years	47	(56)	14	(46)	33	(59)		
Number of long COVID symptoms reported per children, *n* (SD)^a^	12	(8)	10	(8)	13	(7)	0.11	0.34 [−0.08; 0.79]
Mean symptom duration, weeks (SD)	18	(8)	15	(7)	20	(8)	**0.003**	0.66 [0.21; 1.10]
Long COVID complaints related to an organ system, *n*								
General	77	(87)	30	(91)	47	(84)	0.52	0.1 [−0.11; 0.31]
Neurologic	74	(83)	26	(79)	48	(86)	0.40	0.09 [−0.12; 0.30]
Mental	64	(72)	18	(55)	46	(82)	**0.007**	0.31 [0.09; 0.52]
Cardiovascular	55	(62)	20	(61)	35	(63)	0.86	0.02 [−0.13; 0.23]
Gastrointestinal	53	(60)	18	(55)	35	(63)	0.51	0.08 [−0.13; 0.29]
ENT-O	49	(55)	13	(39)	36	(64)	**0.028**	0.25 [0.03; 0.46]
Musculoskeletal	40	(45)	13	(39)	27	(48)	0.51	0.09 [−0.12; 0.29]
Pulmonary	30	(34)	10	(30)	20	(36)	0.65	0.06 [−0.15; 0.26]
Dermatologic	23	(26)	12	(36)	11	(20)	0.13	0.19 [−0.02; 0.40]
Reproductive^b^	11	(12)	0	(0)	11	(20)		
Occurrence of long COVID symptoms after acute COVID-19 disease, *n*								
Continuously present	58	(65)	25	(76)	33	(59)		
1–2 weeks later	9	(10)	3	(9)	6	(11)		
3–4 weeks later	13	(15)	3	(9)	10	(18)		
>1 month later	9	(10)	2	(6)	7	(12)		
Quality of long COVID complaints, *n*								
Completely new complaint	73	(82)	26	(79)	47	(84)		
Change in the intensity/ quality	10	(11)	3	(9)	7	(12)		
Not clear	6	(7)	4	(12)	2	(4)		

*ENT-O* ear–nose–throat and ophthalmology.

Significant differences are presented in bold.

^a^The number of reported symptoms could not exceed 50.

^b^Only two questions addressed the complaints in the reproductive system: if they had erectile dysfunction, and if they had dysmenorrhea, and only dysmenorrhea occurred. Therefore, the comparison of the gender distribution in this category is biased.

Before their first visit to our clinic, 83 (93%) children were screened for laboratory evidence of COVID-19, and 80 (83%) tested positive. Those children who did not have a confirmed diagnosis before, 9 tested positive for SARS-CoV-2 IgG antibodies at our clinic, leading to a total of 89 (100%) with a confirmed SARS-CoV-2 infection (for further details, see Supplementary Table S2).

Overall, 84 (94%) children had a mild or asymptomatic acute COVID-19 illness, while 5 had a moderate acute disease based on the WHO’s classification (https://www.who.int/publications/i/item/global-covid-19-clinical-platform-case-report-form-(crf)-for-post-covid-conditions-(post-covid-19-crf-)). Details of symptoms, the needed medical care during acute COVID and details of medical history can be found in Supplementary Table S3. None of the patients had been vaccinated for SARS-CoV-2 at the time of their first visit.

### Persisting symptoms

**Fig. 1:**
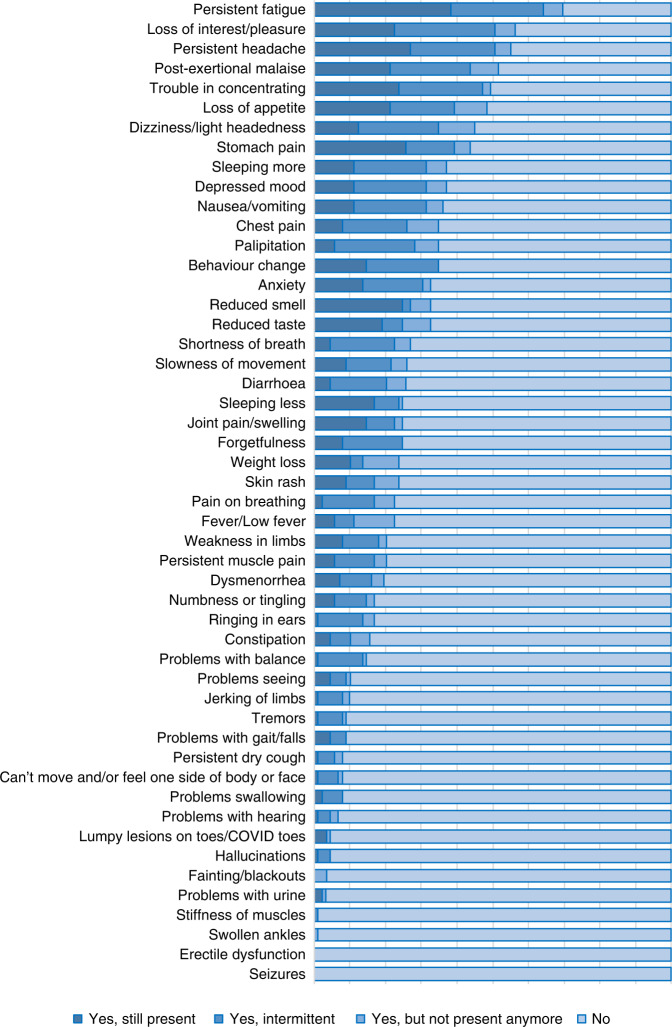
The answers given to the inquired 50 symptoms in the order of incidence. The hierarchy of symptoms is presented according to the total percentage of positive answers (“Yes, still present”, “Yes, intermittent”, and “Yes, but not present anymore”).

**Fig. 2:**
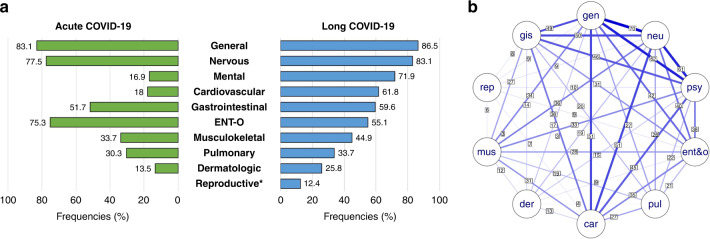
Patterns of COVID-19 related symptom categories in children. **a** Comparing the frequency of symptoms categorized by organ systems during and after acute COVID. *The questionnaire regarding acute symptoms did not contain questions about the reproductive system. **b** Symptoms network graph: the connection lines represent the frequencies of two variables presented simultaneously. gen general, neu nervous, psy mental, ent&o ENT-O (ear, nose, throat, and eye), pul pulmonary, car cardiovascular, der dermatologic, mus musculoskeletal, rep reproductive, gis gastrointestinal.

The average number of symptoms per individual was 12 (ranging from 1 to 33), with the leading complaint—persistent fatigue—occurring in 70% of the children (Fig. 1 and Table 4). Of the 10 organ system categories, an average of 5 ± 2 were affected per person. Similar to acute COVID, general and neurologic complaints were dominating, though mental and cardiologic complaints appeared more frequently in LCS than in acute infection (Fig. 2a and Table 4). The frequently occurring symptoms also showed a tendency to co-occur (Fig. 2b).

At the time of the first visit, symptoms had been present for an average duration of 17.8 weeks (ranging from 5 to 37 weeks); age and duration of symptoms showed a significant positive correlation (*r*(87) = 0.58 [0.42; 0.7], *P* < 0.001). In 58 (65%) children, at least one symptom was continuously present during and after the acute COVID sequel, and in only 9 cases the LCS-related symptoms arose more than a month after the acute disease. In 73 children, there was at least one new, intense symptom that the patient had never experienced before their acute infection; 10 children had symptoms that worsened in their intensity and/or quality, and 6 patients gave uncertain information about their complaints (Table 4).

### Physical and specific findings, laboratory markers

During the physical examination, 45 (51%) children had alterations in their status. Mostly minor findings were observed, mild abdominal tenderness being the most frequent (Supplementary Table S4).

Laboratory examinations showed alterations mostly of low clinical importance or that were hard to interpret because of the lack of other supporting findings (Table 2). However, blood testing alone led to the suspicion of 10 (12%) autoimmune-thyroiditis with specific autoantibodies detected, 4 of whom also had elevated thyroid-stimulating hormone (TSH) values. We diagnosed thyroiditis in 7% of all cases based on the recognition of elevated autoimmune antibody levels followed by positive ultrasound results. (Tables 2 and 3 and Supplementary Table S5).

We indicated at least one imaging examination for 71 children and found alterations in 28% (Table 3 and Supplementary Table S5).

We performed a complete 6-min walk test in 29 cases and more than half (20/29, 67%) of patients reported some complaints during the test, although only 10 (34%) had unquestionable difficulties during the exercise (had to stop, had a drop in SpO_2_ < 95%, or heart rate elevated to 1.5× of their resting heart rate) (Supplementary Tables S5 and S6).

In the 74 children with neurologic complaints, neurologic examinations had non-physiological results in only 7% (5/74) but also revealed a case of trigeminal cephalalgia with polyneuropathy. Overall, 28% of children had hypo/dysosmia with hypo/dysgeusia, and 4% had hypo/dysosmia alone. Seventy-two percent of the children reported having mental problems at some point during their disease; a consultation was necessary in 24 cases, resulting in 15 (23%) new preliminary diagnoses, including major depression and anxiety. Of the 55 children with cardiac complaints, 44 had cardiology-directed examinations of which only 15% (8/55) gave a positive result, though one case of recent myocarditis and one of long-QT syndrome were revealed. Respiratory complaints occurred in 30 children of whom 11 had positive results (37%). Complaints of the GI tract were reported in 53 children, and we indicated 37 GI-oriented examinations and found alterations in 15% (8/53). Dermatologic complaints occurred in 23 children at some point during their disease, and we indicated 8 consultations with dermatologists leading to 5 novel findings, including one Henoch–Schönlein syndrome.

On average, in 25% of the cases we were able to detect at least minor clinical alterations. (Table 1). We detected physical findings that could be directly connected to the linked complaint category in an average of 18% per category. We found the greatest proportion (38%) of connected physical alterations with the specific complaint in the respiratory category (Table 1).

Additional information on our specific findings is available in Supplementary Tables S4 and 5.

### Treatment

As there is no specific treatment available for LCS, in most cases we could only give non-specific supportive therapy for most symptoms. Within the framework of complex, individualized rehabilitation, we offered NSAID for pain, physiotherapy for musculoskeletal problems, Transcutaneous Electrical Nerve Stimulation (TENS) for tension headache, breathing exercises for respiratory disturbances. We also highlighted the beneficial effects of having a scheduled daily routine with regular physical activity and relaxation.

We suggested performing a 12 week long smell training with different types of fragrance oils of choice practiced two times daily for patients with dysosmia or anosmia. In such cases, we frequently observed the complete or partial refusal of meat or other essential nutrient consumption. A short dietary advice handbook was created by a professional dietitian for whom the execution of a healthy, balanced diet seemed to be problematic. It lists multiple alternative nutrient sources, suggests web based and mobile applications for exploring different cooking techniques and approaches which could help masking the disturbing elements of meals. To create a personalized meal plan, an in-person meeting was also available with the dietitian, particularly for those with weight loss. Initiation of transient formula feeding was necessary in two severe cases.

Children with definite diagnoses were treated according to pediatric protocols, as L-Thyroxin therapy was started in two children, inhaled corticosteroids were prescribed in 7 cases, and montelukast in one child.

### Functioning

**Fig. 3:**
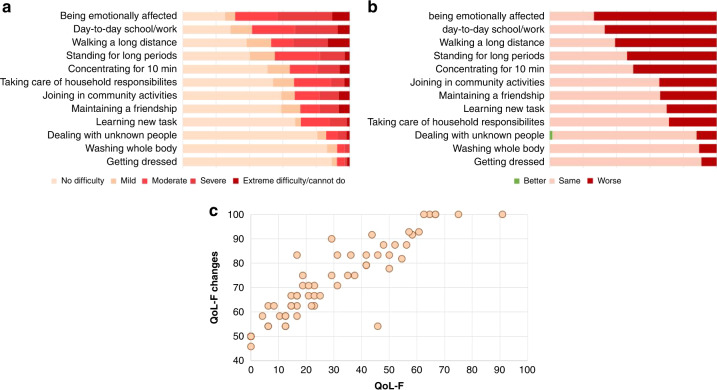
The reported quality of life of children with LC. **a** The QoL-F of children, measured by the 12 items on a 5-grade scale. **b** The QoL-F of children compared to their situations before COVID-19 on a 3-point scale. **c** Association between the unbiased indexes of QoL-F (Difficulties in functioning) and QoL-F changes (Changes compared to pre-COVID-19 status).

There was a total of 53 participants who completed the questions without missing data; we also included those who answered at least half of the questions (n = 14). The total score of QoL-F was between 0 and 90.9 (*M* = 27.1, SD = 22.8) which means mostly mild difficulties in functioning. The total score of QoL-F changes was between 45.8 and 100 (*M* = 70.4, SD = 16.3) meaning that deterioration was most commonly reported, compared to the period before COVID-19. Overall, 14 participants (26%) experienced both at least moderate (QoL-F ≥ 50) difficulties and a worsened status in functioning since COVID-19. The most frequently reported difficult and/or worsened functions were: being emotionally affected by health problems, walking a long distance, day-to-day school/work, standing for long periods, and concentrating for 10 min (Fig. 3a–c).

We asked a separate question about the ability to self-care. Of the 73 who answered, 21 (29%) patients reported an impaired function, while 52 (71%) experienced no change.

## Discussion

Our study involving 89 pediatric LCS patients from Hungary describes the clinical characteristics of a newly emerged, virus-associated chronic condition impairing children’s quality of life for months.^18^ Based on the NICE guideline’s clinical management guidance, we did a diagnosis of exclusion with multi-disciplinary medical examinations (physical, laboratory, radiological examinations, specialist consultations, etc.) without a control group.

We found their recommendation, i.e., to start screening in children as early as the at the “ongoing symptomatic” phase to sit well with our clinical experience, while in adults, it might be acceptable to start the clinical investigations only from the post-COVID phase (i.e., 3 months after).

Our patients reported a broad spectrum and severity of long-lasting symptoms, with fatigue, loss of interest, and headache being the most common ones. In a pediatric meta-analysis, the three most frequently reported symptoms were similar, except they found “dyspnea’ to be second. Based on five controlled studies, out of 14 investigated symptoms, the only ones with significant risk differences between previously COVID-positive and COVID-negative children were loss of smell, cognitive difficulties, sore eyes, sore throat, and headache.^19^

We observed both entirely new complaints in addition to the previously existing, chronic symptoms whose characteristics (intensity, frequency, and quality) worsened after the acute COVID-19. We found patients with exceedingly numerous complaints and with a duration of symptoms over 9 months at the time of our examination. Since LCS is a newly emerged condition, it is yet to be determined how long these effects can last. Based on 13 studies, the median duration of follow-up was 125 days (IQR = 99–231) after acute SARS-CoV-2 infection.^19^ Our data are similar, with a mean of 125 days (ranging from 37 to 259 days; Med = 130, IQR = 104.5 Q1–Q3 = 70.5–175).

Separating the symptoms of LCS from the ones that arose due to the pandemic situation can be challenging, as it is presumed that public restrictions themselves (e.g., lockdowns, online learning) could cause somatization.^20^ The observed high frequency of mental health complaints in our dataset could at least partly be an indicator of this phenomenon. Further clarification is required to determine which of these symptoms derive from the physiopathology of SARS-CoV-2 infection and which from the psychological effects of the disease burden, worsened by the restrictions of the pandemic.^13^

A notable controlled questionnaire study (CLoCk) showed that post-COVID-like symptoms also arose in the general pediatric population but not as many as in those who had COVID-19.^21^

With complex physical, laboratory, and further targeted examinations, we found a high number of minor abnormalities which had no or questionable clinical connection with COVID-19.

Most of our patients had similar neurologic symptoms as adults, like headache, memory problems, and/or smell or taste disturbances, however, in our cohort, one-third of children had hypo/dysosmia, which is much higher than Behnood et al. assumed.^19^

According to a recent meta-analysis, there is still no standard screening protocol for neurological complaints. For whom the neurological physical examination status proved to be seriously altered (blurred vision, diplopia, ptosis, etc.), we executed acute neuro-specific examinations. Both the two non-physiological head MRI and CSF results were of questionable clinical importance and required no specific therapy. We diagnosed one trigeminal cephalalgia with polyneuropathy with the typical complaints and changes in the neurological physical status.^22^ We offered her physiotherapy and clomipramine, but she refused to take the medicine. For the first three months in the follow-up period her complaints remained to persist. The need for further neurologic and psychiatric care were also communicated.

The observed number of neuro-psychiatric complaints (anxiety, depression, etc.) were also high in our dataset and were similar to those in adults and led to several preliminary diagnoses after consultations with psychologists.^22^ No psychotropic medication was prescribed.

Almost two-thirds of our patients had at least one cardiac (cardio-pulmonary) complaint, such as chest pain, palpitation, or post-exertional malaise. Adults experience similar persisting cardiac symptoms and in some cases, myocarditis, pericarditis, and cardiac fibrosis were detected.^22^ Our initial evaluations consisted of 6mWT, ECG, echocardiography, CRP-, troponin- and pro-BNP levels. Although, we could not find increased troponin levels in any of the children, in some cases, we observed slight elevations in the pro-BNP levels, however, with no clinical consequences. We diagnosed multiple post-viral tachycardias and in one case, the suspicion of a recent myocarditis was raised by echocardiography, however cardiac MRI could not confirm it.

Children with a mild acute disease usually do not develop severe pulmonary manifestations, thus we would not expect high rates of late complications with shortness of breath or persistent dry cough. Surprisingly, we observed respiratory complaints in around 34% of all children presenting in our facility with relatively high rates of positive findings with the 6mWT, chest X-Rays, and lung function tests. In adults, chest CT is a popular diagnostic tool for pulmonary consequences, as pulmonary fibrosis and ground-glass opacities can be observed frequently.^22^ Due to the effect of radiation, CT examination is restricted in the field of pediatrics. Still, at the beginning of our study, with the consultation of pediatric pulmonologists and radiologists, we performed three low-dose chest CTs, all with negative results. After reconsideration, we decided to further avoid these examinations. With the help of lung function tests, we diagnosed abnormalities with therapeutic consequences (inhaled corticosteroids) in seven cases.

Loss of appetite, abdominal pain, and nausea were the most commonly reported gastroenterological symptoms with mild abdominal tenderness being the most frequent physical examination finding among our patients. In most cases, lacking objective findings behind these GI complaints, we gave “post-viral abdominal pain” as a final diagnosis. However, we identified a few newly diagnosed cases of lactose intolerance, dysbiosis, or hepatic steatosis, neither of which we supposed to be in strong connection with the previous COVID-19.

The most frequently noted dermatologic complaints were rashes with a broad spectrum of presentation, extension, and color. We did not see any COVID toe^23^ or angioedema,^22^ but we examined a 3.5-year-old male who had been treated in an ICU 5 weeks before their visit with Henoch-Schönlein syndrome and COVID-19 infection, who was still on steroid therapy.

In our dataset, the ratio of symptom-related positive findings was low (a mean of 18% per complaint group), which could mean, that either the rate of somatization was high or that the presumed pathological effects of SARS-CoV-2 could not always be validated by currently available standard medical assessment techniques. Still, in a small but significant proportion of children, we could diagnose ultrasound-confirmed autoimmune thyroiditis, or obstructive respiratory disorders based on lung function test results. Until we know more about the long-term pathological effects of COVID-19, we need to perform a diagnosis of exclusion and cannot make a decision about a causative relationship. Adult case reports of Hashimoto’s thyroiditis and Graves’ disease^24^ after COVID-19 infection are present in the literature. It is known that viral infections can provoke or accelerate different autoimmune diseases and direct damage of SARS-CoV-2 through the ACE-2 receptor is hypothesized as well.^25^ This can also be true for all our outcomes, as SARS-CoV-2 can affect nearly all organ systems.^6^

Several mechanisms are hypothesized to lie behind LC symptoms, including viral cell damage, endothelial and microvascular damage, dysregulation of the immune system, hyperinflammation, hypercoagulational state with thrombosis, and the maladaptation of the ACE2 receptor.^6^ This pathomechanism may overlap with those of SARS and MERS virus; other opinions say that there is a resemblance with the post-Ebola or post-Chikungunya symptoms.^26^

Until LCS stays a diagnosis of exclusion, due to the complexity of symptoms, the length of examinations can be overwhelming for patients and their families both mentally and physically. Evidence-based pediatric guidelines built on controlled studies could aid to rationalize the list of recommended examinations.

Considering changes in the quality of life of children with LC, we mainly detected a long-lasting mild decline after the infection, although outstanding impairments were also observed. It should be noted that this deterioration in QoL scores and many of these symptoms can also be present in the general pediatric population. In the CLoCk study, no difference was found in the distribution of mental health scores and well-being between those who tested positive and negative for coronavirus. Regarding their health-related quality of life, children who previously had COVID-19 were more likely to report problems with mobility, doing usual activities, pain or discomfort, and were more likely to be worried or sad than those who had not.^21^ Further controlled studies are needed to separate the effect of the pandemic era from the infection itself.

As there is no universal evidence-based treatment available for children with LCS, we could only offer non-specific supportive therapy, as part of individualized complex rehabilitation based on NICE guidance.^7^ In some cases, we were able to provide specific therapeutic approaches, like smell training for dys- or anosmia, L-thyroxin for hypothyroidism, inhaled corticosteroids for obstructive diseases.

According to the literature, the acute illness remains hidden or invalidated by laboratory tests in a significant proportion of children, therefore proving a previous SARS-CoV-2 infection can be challenging.^5^ In contrast, we observed that 90% of children were proven with a laboratory test to have had a previous illness by the time they visited our clinic. It is noteworthy that the majority of the LCS children had a mild acute COVID sequel, similar to Ashkenazi et al.’s findings.^8^

Our sample displays an almost 2:1 female-to-male ratio, with females having more LC symptoms. Stephenson et al. and Behnood et al. reported females, older children, and those with mental or physical comorbidities as risk factors for having more symptoms.^19,21^ Most of the children presented in our clinic were school-aged, consistent with recent British,^27^ and Israeli reports.^8^ It is questionable, however, whether younger children also experienced lingering symptoms, being less able to verbalize them efficiently.

Our study lacks a control group, which makes our results non-comparable to an average pediatric population regarding symptoms, quality of life, laboratory results, and other findings, therefore, our goals cannot exceed the detailed description of pediatric LCS. We should also note that families’ perceptions and recollections of the pre-pandemic condition can bias the answers given to our survey. Also, the described duration of symptoms could be influenced by the opening date of our outpatient clinic as well as our limited capacity. The appointment registration process was executed online primarily, and this may have restricted the opportunity for some families. However, supported by the systematic data recording and in person multi-disciplinary assessment we could provide valuable clinical data for future investigations and clinicians examining children with LCS.

In conclusion, we could diagnose clinically important conditions with therapeutic consequences, but the percentage of those findings was low. The most probable, strongly COVID-19-related conditions were the frequently observed olfactory and taste disturbances. The observed concerningly high proportion of mental health problems adds to the burden of the disease. We had patients ranging from those who essentially reported no disturbances in their quality of life, to others having 33 different complaints at a time, with the inability to even dress. It was therefore utterly complicated to reflect the true variability of described complaints and clinical picture in numbers without predefined guidance. Our impression is that setting up complaint-centered outcome measures might be a useful option for better interpreting the severity of the disease. It would also be highly important to establish evidence-based pediatric guidelines, potentially allowing a cost optimization by narrowing down the recommended list of examinations. Our results, however, seem to support that some formerly suggested examinations such as thyroid screening^28^ be retained in future guidelines, as we found (occasionally asymptomatic) autoimmune thyroiditis in 7% of all cases. Until larger, controlled prospective analytical studies are conducted in the pediatric population, it remains ambiguous to rationalize the essential parts of basic checkups, which currently sets a heavy burden on both the healthcare system and patients. Currently, there is no specific treatment option for LCS. A case-control study showed a significantly reduced frequency of LCS in fully vaccinated adults compared to unvaccinated controls.^29^ Therefore, trials approving vaccination in children starting from 5 years of age might open new opportunities for the prevention of LCS in children.

## Supplementary Information

Long_COVID_Supp_clean_version

Supp_Table_S5

Figure S1_300

## Data Availability

The datasets generated during and analyzed during the current study are available from the corresponding author on reasonable request.
