# Supplementary material for: Clinical assessment of children with long COVID syndrome
Source: Pediatr Res. 2022 Dec 7;93(6):1616–25. doi: 10.1038/s41390-022-02378-0 (PMC10172119; doi:10.1038/s41390-022-02378-0)
Supplement: Supplementary file 1 — Long_COVID_Supp_clean_version [file 41390_2022_2378_MOESM1_ESM.pdf]

Clinical assessment of children with long COVID syndrome
Supplementary Appendix

Table of Contents

Page 1

1. Table S1 — Page 2
2. Table S2 — Page 3
3. Table S3 — Page 4
4. Table S4 — Page 5
5. Table S5 — Page 6
6. Table S6 — Page 9
7. Figure S1 — Page 10

Table S1. List of the 50 Long COVID Complaints Inquired in the Online Survey and the Corresponding Organ-System Categories.

**GENERAL**	**GASTROINTESTINAL**
Fever, low fever	Constipation
Loss of appetite	Diarrhoea
Persistent fatigue	Nausea/ vomiting
Weight loss	Problems swallowing
**NEUROLOGIC**	Stomach pain
Can't move and/or feel one side of body or face	**EAR-NOSE-THROAT & OPHTALMOLOGIC**
Dizziness/light headedness	Problems with hearing
Fainting/ blackouts	Reduced taste
Forgetfulness	Problems seeing
Tremors	Reduced smell
Numbness or tingling	Ringing in ears
Persistent headache	**MUSCULOSKELETAL**
Problems with balance	Joint pain/swelling
Seizures	Persistent muscle pain
Slowness of movement	Swollen ankles
Weakness in limbs	Pain on breathing
Problems passing urine	Jerking of limbs
Sleeping less	Problems with gait/ falls
Sleeping more	Stiffness of muscles
**MENTAL**	**PULMONARY**
Anxiety	Persistent dry cough
Behaviour change	Shortness of breath
Depressed mood	**DERMATOLOGIC**
Loss of interest/ pleasure	Lumpy lesions on toes/ COVID toes
Trouble in concentrating	Skin rash
Hallucinations	**REPRODUCTIVE**
**CARDIOLOVASCULAR**	Dysmenorrhea
Chest pain	Erectile dysfunctions
Palpitations	
Post-exertional malaise	

Table S2. COVID Testing in Long COVID Children Before and After Their First Visit at Our Clinic*

Time of testing		Tested Positive (n)	*(%)*
Tested before the first visit at our clinic[ǂ]	Total	80/83	*(96)*
	PCR	48/55	*(87)*
	RAT (LFD)	15/32	*(47)*
	IgG	26/27	*(96)*
Tested at the first visit			
	IgG	78/88	*(89)*

* PCR denotes Polimerase Chain Reaction, RAT rapid antigen test, LFD lateral flow device, and IgG denotes Anti-SARS-CoV-2 IgG

[ǂ] The total sum of patients may exceed 88 since one child could be tested with more than one method.

Table S3. Medical History of the 97 Children with Long COVID

Characteristics	Overall, n	(%)	Male, n	(%)	Female, n	(%)
Children included	89		33		56	
**Acute COVID disease**						
Severity						
Mild	84	(94)	31	(94)	53	(95)
Moderate	5	(6)	2	(6)	3	(5)
Severe	0	(0)				
Level of medical care needed						
none	34	(38)	10	(30)	24	(43)
homecare / telemedicine	41	(46)	17	(52)	24	(43)
ambulatory care	11	(11)	5	(15)	6	(11)
hospital admission	3	(3)	1	(3)	2	(3)
Medication given during the acute COVID						
None	60	(67)	24	(73)	36	(64)
Yes	29	(33)	9	(27)	20	(36)
Antibiotics	11					
Corticosteroids	3					
Medical history at the first visit						
Pre-existing conditions						
Total, including allergies	37	(42)	13	(39)	24	(43)
Total, without allergies	24	(27)	8	(24)	16	(29)
Allergies	25	(28)	10	(30)	15	(27)
Chronic Lung disease	7	(8)	4	(12)	3	(5)
Digestive disease	6	(7)	0	(0)	6	(10)
Mental Health conditions	6	(7)	4	(12)	2	(4)
Endocrine/metabolic diseases	4	(4)	0	(0)	4	(7)
Autoimmune thyreoiditis	*1*	*(1)*	*0*	*(0)*	*1*	*(2)*
Diabetes mellitus (Type I)	*1*	*(1)*	*0*	*(0)*	*1*	*(2)*
Neurologic disorders	3	(3)	1	(3)	2	(4)
Immune System disorders	2	(2)	0	(0)	2	(4)
Musculoskeletal diseases	1	(1)	0	(0)	1	(2)
Gynaecologic diseases	1	(1)	-	-	1	(2)
Was currently taking medications	26	(29)	10	(30)	16	(29)
Coronavirus vaccination (before the first visit)	0	(0)				

Table S4. List of physical findings

Physical finding	n	
mild abdominal tenderness	17	19,1
obesity	5	5,6
pharyngeal erythema	5	5,6
cervical lymphadenopathy	3	3,4
dizziness	3	3,4
tachycardia	3	3,4
marbled skin	2	2,2
rash	2	2,2
skin discolouration	2	2,2
strias	2	2,2
systolic cardiac murmur	2	2,2
acnes on skin	1	1,1
asthenia	1	1,1
blurred vision	1	1,1
bradykinesia	1	1,1
diplopia	1	1,1
hip arthralgia	1	1,1
muscular weakness	1	1,1
musculosceletal chest pain	1	1,1
nystagmus	1	1,1
occular discomfort	1	1,1
onychoclasis	1	1,1
paresthesia	1	1,1
pharyngeal erythema	1	1,1
powerless/feeble	1	1,1
purpura	1	1,1
renal pain	1	1,1
Romberg test positive	1	1,1
skin mass	1	1,1
skull tenderness	1	1,1
tachypnoe	1	1,1
unilateral abnormal breath sounds	1	1,1
unilateral ptosis	1	1,1

Table S5. List of abnormal findings (See separately in the Excel file named: „Supp_Table_S5")

Table S6 – Results of the 6 minute walk test

Patient	Presence of symptoms (yes/no)	6mWT symptoms 1 - subjective 2 - had to stop (objective symptom)	Meters walked	Pulse at rest (beat/minute)	Saturation at rest (%)	Pulse at the end (beat/minute)	Saturation at the end (%)
1	Yes	1	94	88	99	106	93
2	No		512	0	0	103	99
3	No		490	0	0	93	99
4	Yes	1	300	79	98	97	99
5	No		420	105	99	0	90
6	Yes	1	395	83	99	91	98
7	Yes	1	500	115	99	148	96
8	No		490	104	97	150	98
9	No		450	100	98	0	99
10	No		583	110	98	174	99
11	No		560	103	99	140	99
12	No		396	90	99	110	97
13	Yes	1	452	75	97	142	98
14	No		424	89	99	124	97
15	Yes	1	0	98	99	147	97
16	Yes	1	450	115	99	164	97
17	No		338	77	98	86	98
18	Yes	1	556	105	98	128	98
19	Yes	1	336	101	98	113	99
20	Yes	1	400	100	99	113	97
21	No		425	92	99	122	98
22	Yes	1	315	98	99	105	99
23	No		525	63	98	132	97
24	Yes	2	504	88	99	107	99
25	Yes	1	616	71	98	123	91
26	Yes	1	384	138	99	152	98
27	No		320	93	99	153	98
28	No		550	67	98	130	97
29	Yes		425	97	98	147	98

Figure S1. The frequency of complaints in organ categories and connected physical findings (see in separate "Supp_Figure_S1")
