# Supplementary figures and images for: Clinical assessment of children with long COVID syndrome
Source: Pediatr Res. 2022 Dec 7;93(6):1616–25. doi: 10.1038/s41390-022-02378-0 (PMC10172119; doi:10.1038/s41390-022-02378-0)

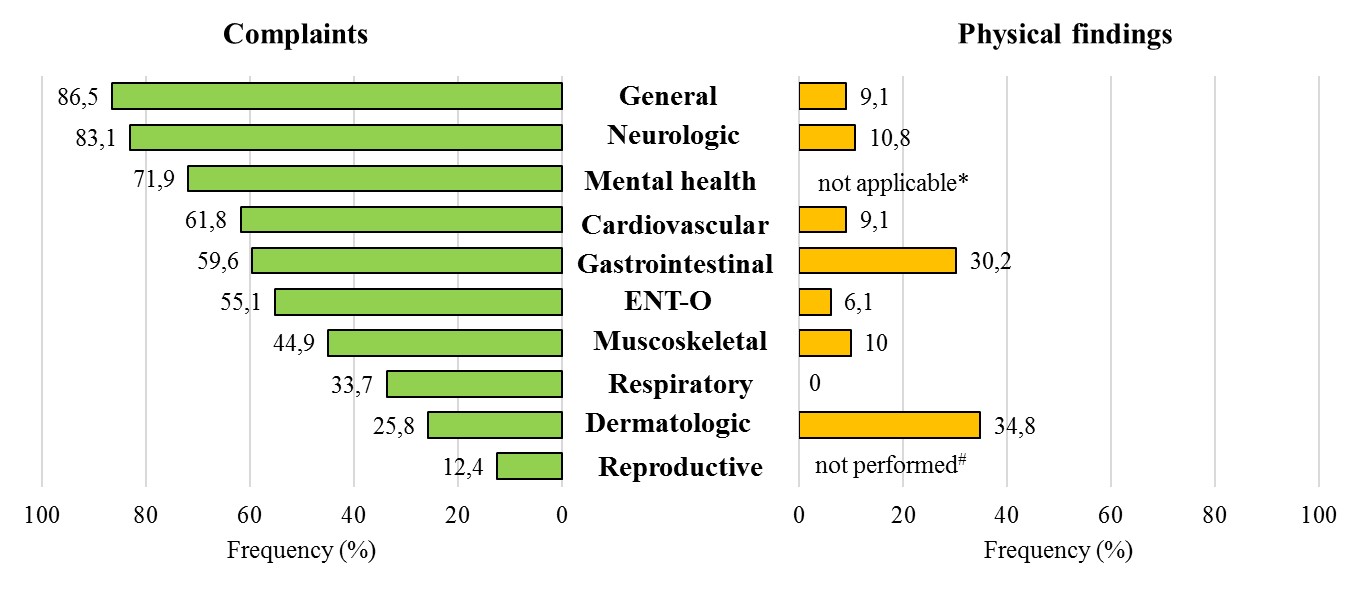

Supplement: Supplementary file 3 — Figure S1_300 [file 41390_2022_2378_MOESM3_ESM.jpg]
